# Superfluous Spectacles

**Published:** 1894-03

**Authors:** James Moores Ball

**Affiliations:** Keokuk, Iowa


### "Superfluous Spectacles."

KEOKUK, IOWA, FEB. 24, 1894.

*To the Editor:*—Superfluous spectacles are those prescribed by incompetent persons. Who are they? Surely not the educated physician who, after years of preparation, drops into ophthalmology by a sort of natural affinity; surely not the man or woman, who, when examining the eye, sees not only an optical apparatus but a living being behind it. No. The incompetents are those half-hatched and abortive products ground out by the so-called "ophthalmic colleges." These "colleges" are engaged in manufacturing opticians out of jewelers and oculists out of jays. Six weeks' time suffices in either case to produce a diploma-bearing individual, who goes forth to prey upon the credulity of the populace. These are the persons who are to blame for "superfluous spectacles."

Every educated physician engaged in ophthalmic practice knows that many cases of asthenopia, in which refractive error exists, are not cured by glasses. The glasses are blamed, the oculist denounced, and regular medicine is brought into disrepute, all because too much confidence has been placed in the curative power of the lenses. In these very cases, glasses are needed because of the existence of error. The fact that asthenopic symptoms are not relieved by lenses should not lead us to regard such lenses as "superfluous spectacles." Glasses will cure many strange symptoms—but they are not omnipotent. Often change of air, of occupation or the treatment of nasal disease; often the use of tonics and other constitutional treatment will be necessary. It is in such cases that the "eye oculist" of six weeks' preparation will fail.

What is the remedy? *Abolish the "Ophthalmic Colleges."* If they are valuable, why not establish rectal, genito-urinary, hepatic, stomachic and pancreatic colleges? Specialism in medicine has gone mad. JAMES MOORES BALL, M.D.

—*Journal American Medical Association*

